# Primary lymphoma of the lumbar vertebrae: a case report and review of the literature

**DOI:** 10.1186/s13256-022-03725-9

**Published:** 2023-02-12

**Authors:** Saeid Safaei, Parisa Azimi, Taravat Yazdanian, Saadat Molanaei

**Affiliations:** 1 Knee and Sport Medicine Research Center, Milad General Hospital, Tehran, Iran; 2 Neuroscience Research Center, Shahid Beheshti University of Medical Sciences, Arabi Ave, Daneshjoo Blvd, Velenjak, 19839-63113 Tehran Iran; 3 School of Medicine, Capital Medical University, Beijing, China; 4 Department of Pathology, Milad General Hospital, Tehran, Iran

**Keywords:** Primary lymphoma, L3 vertebra, Review

## Abstract

**Background:**

Primary lymphoma of the spinal vertebrae (PLSV) is an exceedingly rare disease with an unclear optimal treatment plan. We analyzed the clinical features of PLSV in the patient to strengthen our understanding of the disease and to review the literature.

**Case presentation:**

A 65-year-old Persian man was admitted to our hospital with severe low back pain. The patient underwent radiological examinations including computed tomography (CT) scan, magnetic resonance imaging (MRI), and single-photon emission computed tomography (SPECT). These examinations revealed a lesion in the L3 vertebra. Histological analysis showed a high-grade lymphoma. The patient underwent an L3 corpectomy with expandable cage placement, followed by an L2–L4 lateral screw placement with rod fixation. Also, facetectomy, laminectomy, and total spondylectomy were performed. Pedicle screws were inserted from L1 to L5. Titanium mesh was placed on the post-laminectomy defect. The treatment continued with local radiotherapy and chemotherapy. Post-treatment, the patient showed no new neurological deficit, and in the final follow-up, the patient had achieved a good recovery.

**Conclusion:**

To our knowledge, no prior published literature has revealed a primary lymphoma of the lumbar vertebrae. Here, we report this case of PLSV for the first time and provide a brief review of the literature.

## Background

Primary bone lymphoma (PBL) is a rare disease localized to the bone without confirmation of lymphoma in lymph nodes or other parts of the body [1]. This disease was first described by Oberling in 1928 [2]. Most patients with this disease are adults [3]. The prevalence of PBL is estimated at 3–7% amongst primary bone tumors and less than 2% amongst all lymphomas in adults [3]. Only 9% of all PBL has been reported in the spinal column [4]. The exact definition of PBL in medicine is still a controversial issue and much debated [3]. Overall, the diagnostic criteria used for PBL are those established by the World Health Organization (WHO) and International Extranodal Lymphoma Study Group (IELSG) [3]. The treatment of these patients is challenging, and the best results come from early diagnosis. Here we report a case of primary lymphoma of the lumbar vertebrae in an adult. The clinical summary, imaging findings, and surgical procedures are discussed. Also, a review of the literature on primary lymphoma of the spinal vertebrae (PLSV) is presented.

## Case presentation

A 65-year-old Persian man was admitted with severe low back pain. The current symptoms started 1 month before the admission. He experienced muscle weakness (3/5 muscle strength on the Medical Research Council scale) with progressive urinary retention and constipation. Therefore, he developed cauda equina syndrome. The patient did not experience any unintentional weight loss, night sweats, or fever. All routine laboratory tests including complete blood count with differential (CBC w/diff), thyroid function tests (TFTs), liver function tests (LFTs), blood urea nitrogen (BUN), creatinine, alkaline phosphatase, and ferritin levels, and tumor survey were normal.

**Fig. 1:**
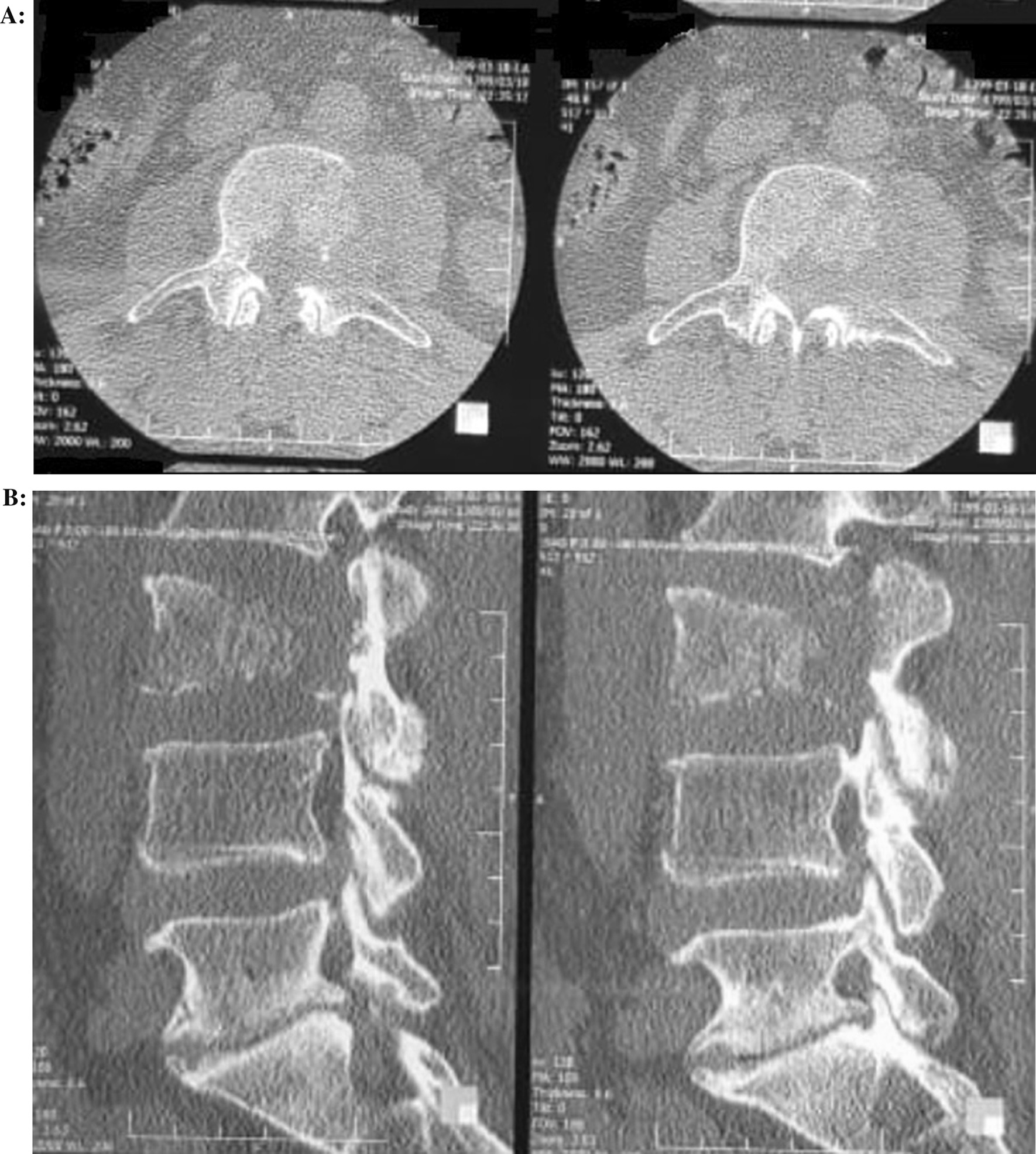
Axial (**A**) and sagittal (**B**) computed tomography images showing bony destruction on L3 vertebra

**Fig. 2:**
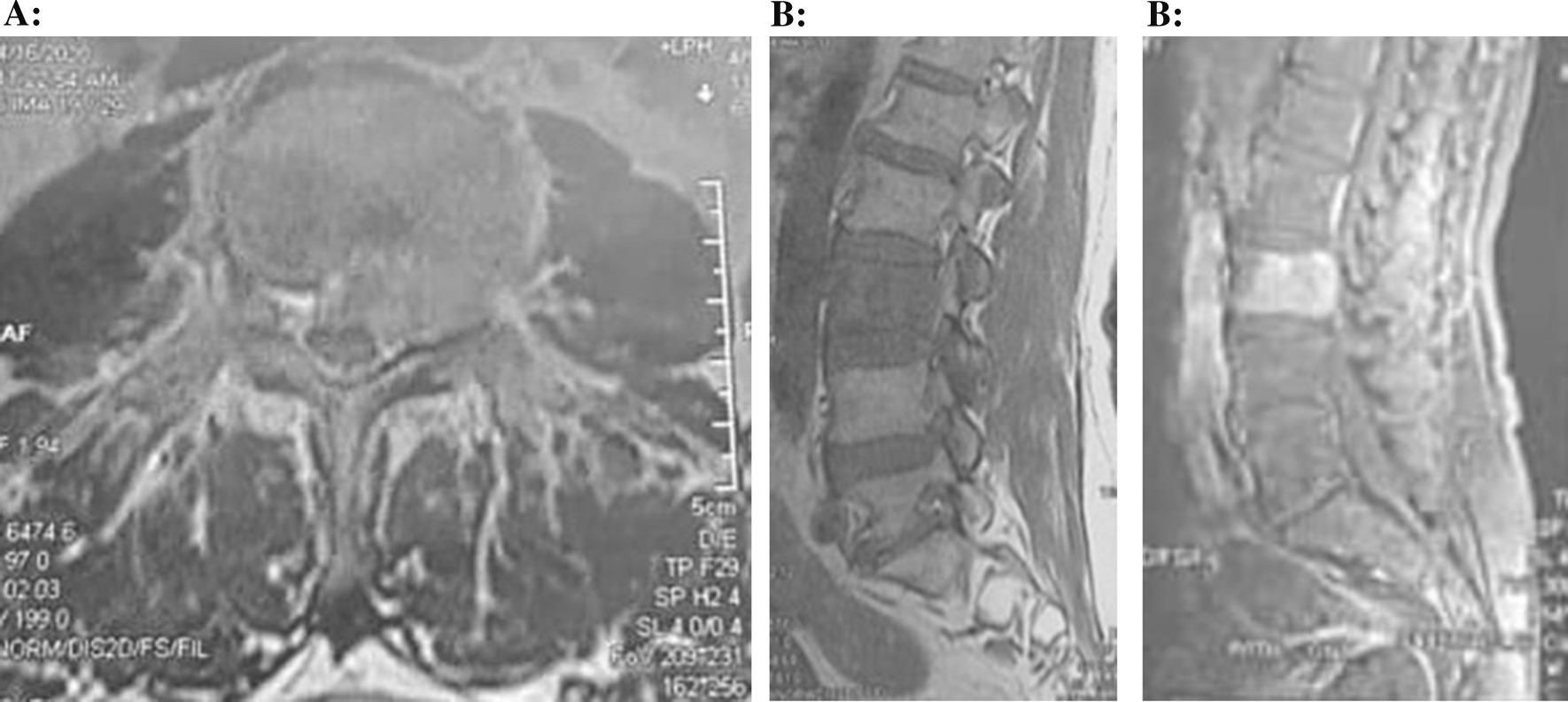
Axial (**A**) and sagittal (**B**) magnetic resonance images showing signal changes and cord compression on the L3 level

Diagnostic radiographic imaging was performed according to symptoms and physical examination signs. Computed tomography (CT) scans of the lumbar vertebrae demonstrated a lesion in the L3 vertebra with destruction of the L3 vertebral body and the left pedicle (Fig. 1). Magnetic resonance imaging (MRI) of the lumbosacral spine revealed a lesion of the L3 vertebral body with hyposignal on T1-weighted and hypersignal on T2-weighted image, with significant compression of the spinal cord. Short tau inversion recovery (STIR) sequences also showed bone marrow changes (Fig. 2). No hemorrhage was seen. A whole-body bone scan [single-photon emission computed tomography (SPECT)] was performed to evaluate various bone-related pathology, and increased absorption was seen only in the L3 vertebra. The diagnosis before surgery was a primary spinal tumor.

**Fig. 3:**
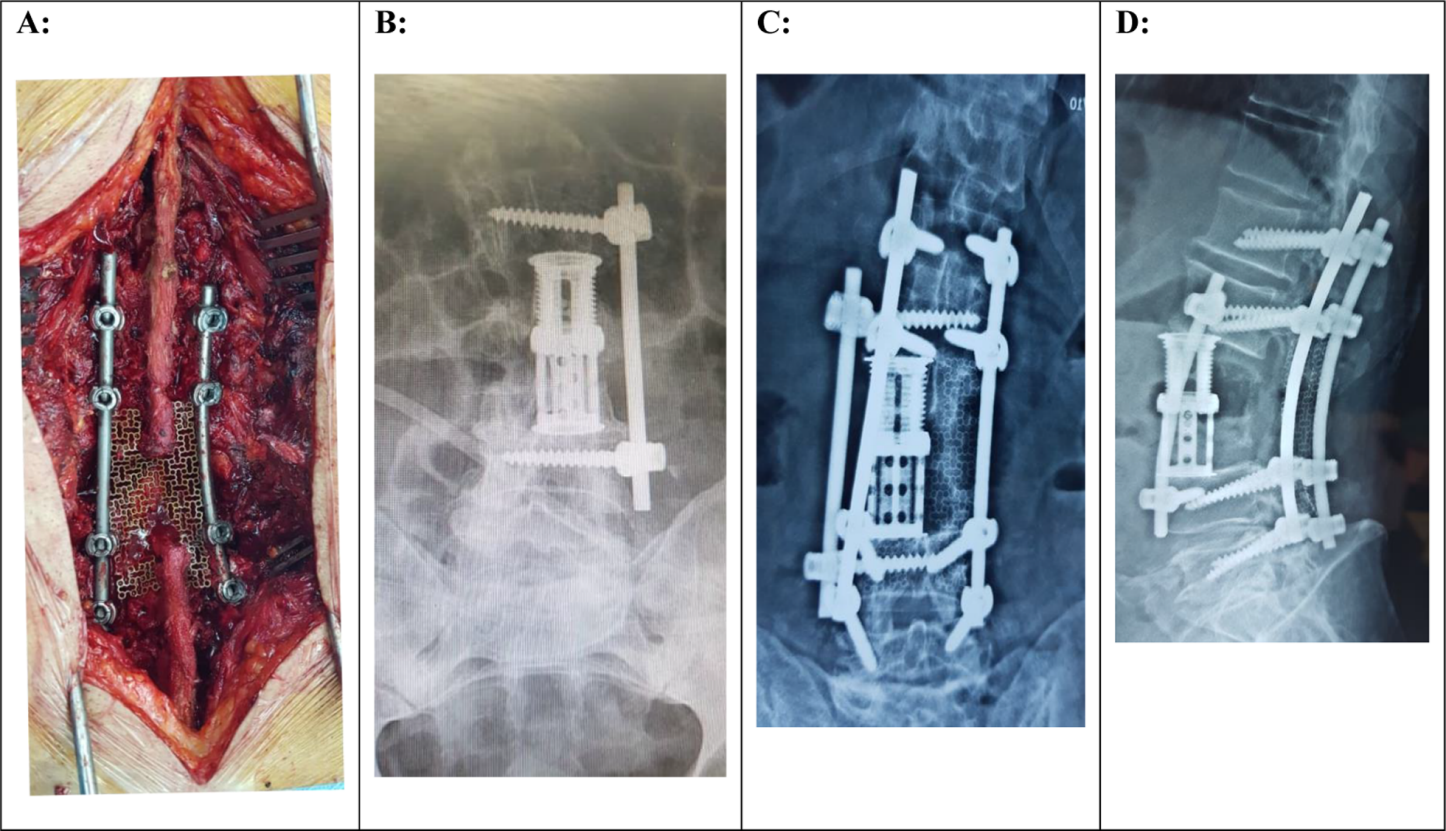
Intraoperative photograph (**A**) showing titanium mesh placed on the post-laminectomy defect. X-ray imaging after first surgery (**B**), anteroposterior and lateral lumbar spine radiographs (**C** and **D**) at the last follow-up

Rapid deterioration of the neurological condition of our patient led to the decision to perform surgery without biopsy. In April 2020, 3 days after the first visit, under general anesthesia, the patient underwent an L3 corpectomy with expandable cage placement, followed by an L2–L4 lateral screw placement with rod fixation (Fig. 3). The vertebral body sample obtained from the surgery was submitted for pathology examination. Three days after the first surgery, facetectomy, laminectomy, and total spondylectomy were performed with pedicle screw from L1–L5. A titanium mesh was placed over the laminectomy site to protect the spinal cord and fusion with autograft bone from the patient's iliac crest (Fig. 3).

**Fig. 4:**
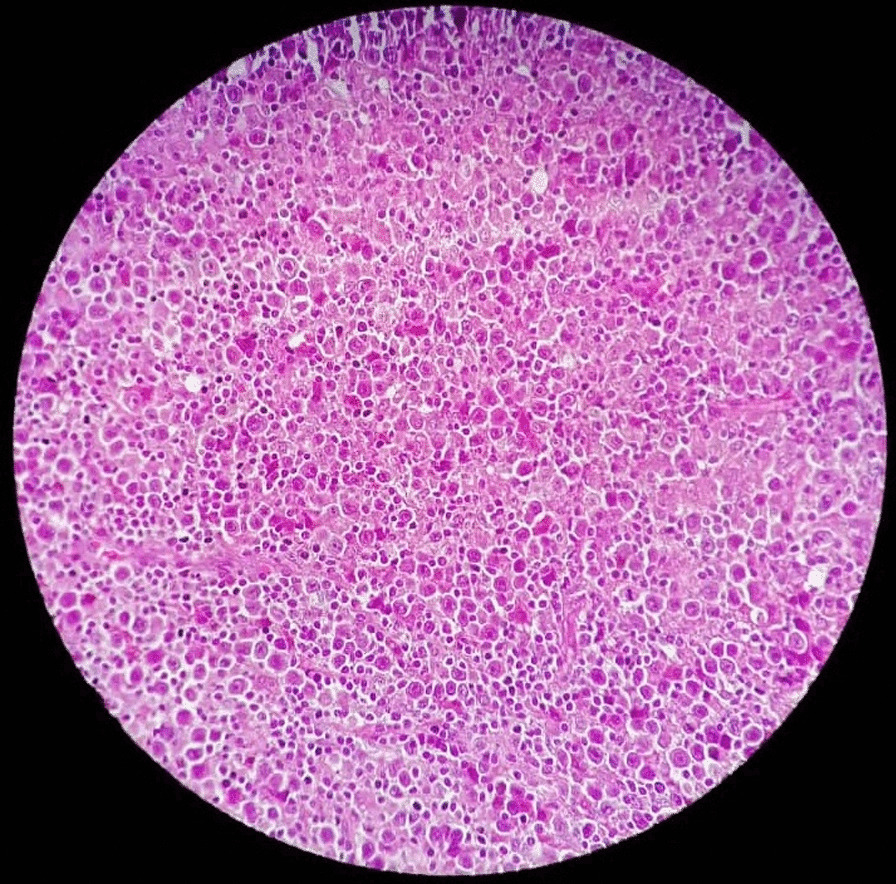
Histopathology examination of the patient

Excisional biopsy revealed high-grade lymphoma (Fig. 4). Histopathology and diagnosis confirmed diffuse large B-cell lymphoma (DLBCL), which consisted of large lymphoid cells that had vesiculated nuclei with prominent nucleoli and frequent mitotic activity. Small mature lymphoid cells with mature nuclei were also found. Immunohistochemical staining was cluster of differentiation 3 (CD3)-positive for non-neoplastic cells, and CD3-negative, CD15-negative, and CD20-positive for neoplastic cells. Antigen Ki67 was positive in about 70% of tumor cells. Bone marrow biopsy was negative. A staging examination including CT of the thorax, abdomen, and pelvis presented no additional suspicious sites.

Post-operation, the patient showed no new neurological deficit. The treatment was continued with a total of six cycles of R-CHOP chemotherapy (rituximab, cyclophosphamide, hydroxydaunorubicin [doxorubicin], vincristine [Oncovin] and prednisolone) and then local radiotherapy by a clinical oncologist and radiation oncologist, respectively. The patient received a total of 40 Gy over 22 sessions. The intensity of back pain was assessed by visual analog scale (VAS), before and after the surgery. Eight months after surgery (last visit), an improvement was seen in back pain intensity (VAS) and neurological status. Anteroposterior and lateral lumbar spine radiographs are shown in Fig. 3C and D at the last follow-up. Based on clinical status, radiological imaging, and laboratory tests, no extraspinal disease was observed. We reviewed a PubMed database for the following search terms: primary lymphoma, spine, and vertebrae from 2010 to 2020. We selected English, full-text publications which exactly corresponded to our subject of interest. Only primary lymphoma of the lumbar vertebrae without involving other bones was considered. In addition, primary lymphoma of the sacrum was not included in this study. We excluded studies that did not mention the type of lymphoma (Table 1) [4–7].

**Table 1:** Summary of five reported cases of primary lymphoma of the spinal vertebrae

Author (Ref.)	Year	Age/gender	Level	Diagnosis	Treatment	Outcome
Smith *et al*. [4]	2010	23/M	C7	CT + MRI + PET + biopsy test	Surgery + local radiotherapy + chemotherapy	Treatment halted the progression of the primary disease and preserved neurological function
Park *et al*. [5]	2012	27/M	T11	CT + MRI + PET + biopsy test	Surgery + local radiotherapy + chemotherapy	At 1 year after surgery, he was pain-free and able to return to full-time work
Undabeitia *et al*. [6]	2014	73/M	C4	CT + MRI + PET + biopsy test	Surgery + local radiotherapy	Chemotherapy was not applied due to the age and comorbidities of our patient. When neurological deficit appeared, early surgery for decompression was indicated, followed by local radiotherapy and systemic chemotherapy
Jia *et al*. [7]	2017	79/F	T5	CT + MRI + biopsy test	Surgery + chemotherapy	At the 9-month follow-up, the clinical result was still satisfactory
Present study	2020	65/M	L3	CT + MRI + SPECT + biopsy test	Surgery + local radiotherapy + chemotherapy	In the final follow-up, the patient had a good prognosis

*CT* computed tomography, *MRI* magnetic resonance imaging, *SPECT* single-photon emission computed tomography; *PET* positron emission tomography

## Discussion and conclusions

To our knowledge, this case is the first report of primary lymphoma of the lumbar vertebrae with such a clinical presentation. Primary lymphoma arising from the lumbar vertebrae is exceedingly rare. Diagnosing the disease may be challenging due to the rarity and nonspecific clinical-radiological features. Spine surgeons should be aware of the unique presentation of this disease and that combination of surgery, radiotherapy, and chemotherapy is a successful treatment strategy.

Sharma *et al*. [8] presented 49 patients with PBL. Lymphoma involved multiple bones including spine + rib cage (51.0%) (*n* = 25) and pelvis (34.6%) (*n* = 17). No case involved single vertebrae. Zhang *et al*. [9] analyzed 61 PBL cases. Only one case involved a single spinal vertebra. However, the location of the vertebra was not reported. Beal *et al*. [10] reported a series of PBL that included 82 patients. In that series, the frequency of different bone involvement was femur (27%), pelvis (15%), tibia/fibula (13%), polyostotic (13%), humerus (12%), spine (9%) (*n* = 7), other (5%), mandible (2%), radius/ulna (1%), scapula (1%), and skull (1%). Further details of the spinal vertebrae were not reported. To improve overall survival rates of patients, they recommended the use of combined-modality over single-modality treatment, which is in line with this study. However, patients with advanced-stage disease who received chemotherapy alone have a good outcome when compared with those who received chemotherapy plus radiotherapy [11]. In addition, according to the WHO classification, lymphoma involving the bone can be classified into four groups. Group 1 is lymphoma in a single bone position with or without local lymph node involvement [12]. We diagnosed the primary lymphoma of the lumbar vertebrae in our case based on the WHO classification to be primary lymphoma group 1. It should be noted that all treatment decisions will depend on the patient's status. Further studies are necessary to clarify the characteristics of PBL and its optimal treatment strategy.

Diagnosis of PBL may be challenging. Different imaging techniques including CT, MRI, SPECT, and positron emission tomography (PET) combined with biopsy results are used to diagnose this disease. However, a biopsy of an adjacent lymph node or directly from the involved bone forms is the foundation of the diagnosis [4]. In this study, we used CT, MRI, and SPECT imaging combined with biopsy results to diagnose the disease. However, integrated PET/CT was not used, which is a limitation of this study. Similar to this study, on MRI, the lesions often showed hypointensity on T1-weighted imaging and hyperintensity on T2-weighted imaging [13]. The contrast-enhanced images demonstrated areas of enhancement within the lesion [14]. As our surgical approach, corpectomy and decompression with fusion were successful in preventing progressive neurological damage [4, 6]. Also, local radiotherapy and systemic chemotherapy are recommended based on the patient's condition [4–7]. It is interesting that in our case, similar to other cases reported in the literature [4–7], the clinical results show a good prognosis in the final follow-up. This may be because this disease cannot affect any part of the body other than the spinal vertebrae.

PBL is a rare but known entity. The most common site of occurrence is the long bones [15]. Spinal involvement was reported in only 9% of cases, and dorsal vertebrae are the most common site [4, 10]. While isolated lumbar vertebral lymphoma has not been reported yet, it seems that there is no difference between vertebrae with lymphoma involvement, although more study is needed. This case was unique insofar as it is the first report of primary lymphoma of the lumbar vertebrae at the L3 position without involving other bones. Hence, it may have a role to play in medical research and evidence-based medicine. More importantly, it gives an indication of the decision-making process, and thus other clinicians can gain a broader understanding of clinical diagnoses, treatments, and outcomes with respect to their own cases. In the present case, the initial symptoms may have resulted result in lumbar cord compression. We believe that early diagnosis and early surgery for decompression of the spinal cord, followed by local radiotherapy and chemotherapy, was successful in preventing progressive neurological damage and maintaining quality of life.

## Data Availability

All the data supporting our findings are contained within the manuscript.

## Abbreviations

PET: Positron emission tomography
PLSV: Primary lymphoma of the spinal vertebrae
SPECT: Single-photon emission computed tomography
